# Interspecific competition with the American *Xanthium orientale* L. as a possible cause of the decline of the Old-World *X. strumarium* L.

**DOI:** 10.1038/s41598-025-17814-4

**Published:** 2025-09-01

**Authors:** Eleonora Manzo, Chiara-Sophie Epifanio, Julius F. Pahl, Salvatore Tomasello

**Affiliations:** 1 Department of Systematics, Biodiversity and Evolution of Plants, Albrecht-Von-Haller Institute for Plant Sciences, University of Göttingen, Untere Karspüle 2, 37073 Göttingen, Germany; 2 Leibniz Institute DSMZ-German Collection of Microorganisms and Cell Cultures GmbH, 38124 Braunschweig, Germany

**Keywords:** Allelopathy, Competitive exclusion, Garden experiments, Interspecific competition, *Xanthium*, Plant ecology, Plant sciences, Ecology

## Abstract

*Xanthium* is represented in Europe by three species complexes: *X. strumarium* L., *X. orientale* L., and *X. spinosum* L. The former two complexes are similar, in both morphology and ecological requirements. *Xanthium strumarium* is native to the Old World, whereas *X. orientale* originates from America and was accidentally introduced into Europe about two centuries ago. Since then, it has colonized the whole continent, while the native congener has become increasingly rare.

Over two years, we conducted competition experiments to assess the impact of the introduced *X. orientale* on the fitness of the native *X. strumarium*. Germination time, dry biomass, number of burs (pistillate flower heads) and bur biomass were measured as proxies of fitness. *Xanthium strumarium* was grown alone (control), together with conspecifics (intraspecific competition) or with *X. orientale* plants (interspecific competition). We also evaluated the allelopathic effect of *X. orientale* over *X. strumarium*, by watering *Xanthium* seedlings with exudate of *X. orientale* dry leaves.

Growth and reproductive traits of *X. strumarium* were significantly lower in individuals growing in proximity of *X. orientale* compared to the control, whereas intraspecific competition has a lower but still significant effect. *Xanthium orientale,* although, germinates and grows faster than the Old-World congener, and under interspecific competition regime, *X. strumarium* produces significantly lower biomass, number of burs and bur biomass. Watering with exudates negatively influences the germination and the growth of the two species. We therefore believe that interspecific competition of the introduced congener may be one of the causes explaining the drastic decline of *X. strumarium* populations in Europe in the past century.

**Supplementary Information:**

The online version contains supplementary material available at 10.1038/s41598-025-17814-4.

## Introduction

Invasive species are widely recognised as major contributors to biodiversity loss^1^. An invasive species can be defined as a non-native species (alien species) whose introduction or spread has been found to threaten or adversely impact biodiversity and related ecosystem services (EU Regulation No 1143/2014, Article 3, clause 2). Invasive species represent a significant conservation challenge, having been responsible for numerous extinctions^2^. Nevertheless, a significant proportion of the evidence supporting this argument is based on straightforward correlations between the prevalence of exotic species and the decline of native species in degraded ecosystems. However, it is important to recognise that direct causality is not the only possible interpretation of these findings^1^.

The concept of competition in plant ecology dates back centuries. Authors such as de Crescentiis in the fourteenth century (*Ruralium Commodorum*) and De Candolle^3^ discussed competitive interactions among plants. Darwin^4^, influenced by Malthus^5^, emphasised competition as a selective force shaping species evolution. Early ecologists regarded competition as an intrinsic part of nature^6^.

The principle of competitive exclusion, as defined by Hardin^7^, states that “complete competitors cannot coexist”. This implies that, in case two non-interbreeding populations occupy the same ecological niche and geographic territory, one will inevitably exclude the other. Grime^8^ identified several characteristics that contribute to the success and competitiveness of herbaceous plants. These include a tall stature, a growth form that allows for effective utilisation of the environment below and above ground, a high maximum potential relative growth rate, and a tendency to deposit a dense layer of litter on the ground surface.

Few studies explicitly compared co-occurring close relatives among plant species, estimating differences in both niche and competitive ability^9^. It is hypothesised that species that are part of the same genus and have the same life form can exhibit similarities in resource capture. Garcia-Serrano et al.^10^ conducted a comparative analysis of the competitive interactions between three species of the genus *Senecio* L.: one native and two invasive. Their findings revealed that in the majority of cases, one of the invasive species exhibited a higher competitive ability, leading to a greater population density. There are various studies that have proven the reduction of native populations due to competition with invasive congener, both in animal and plants (e.g. *Senecio*^10^; *Podarcis*^11^; *Natrix*^12^).

**Fig. 1:**
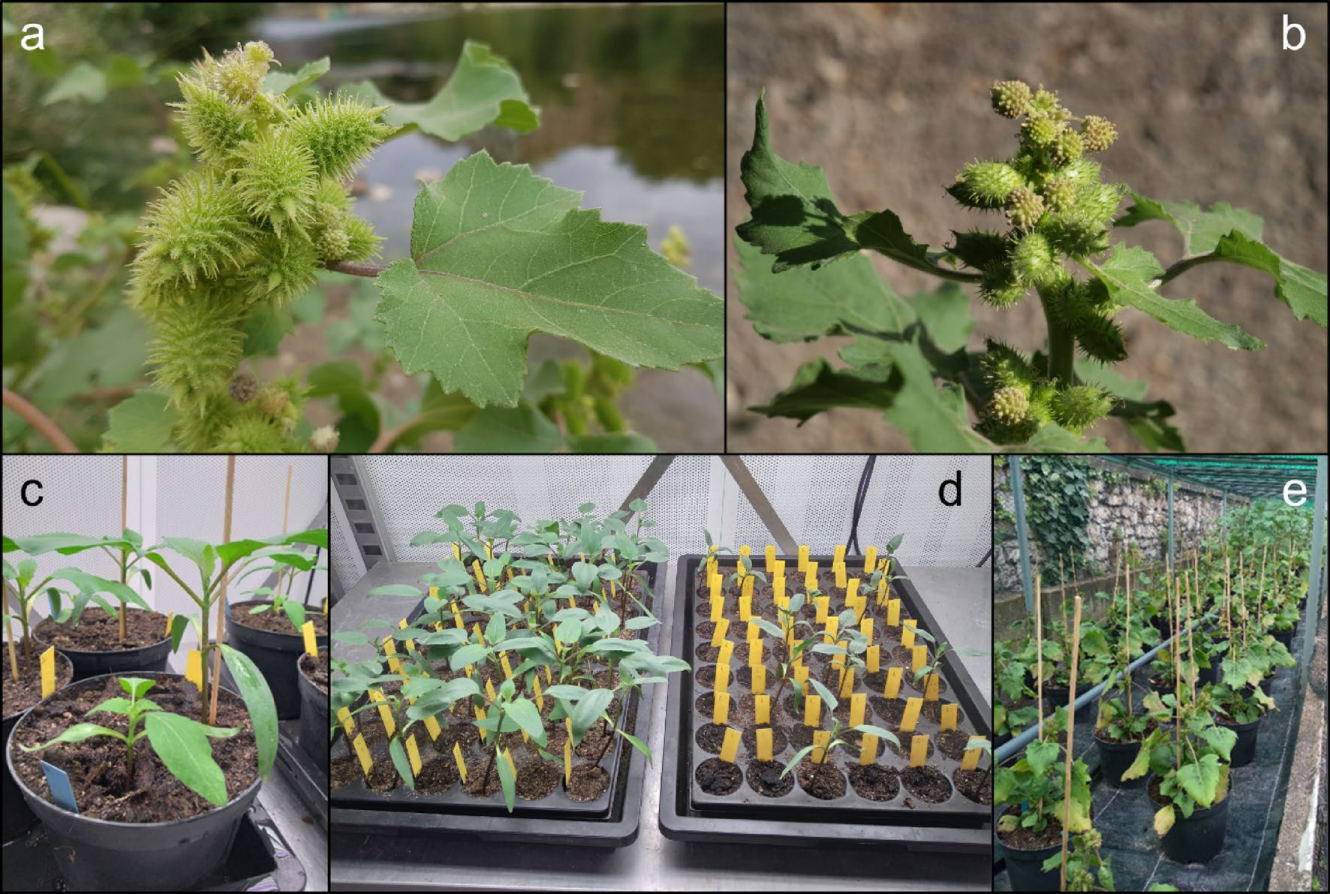
(**a**) Details of the burs of a *Xanthium orientale* subsp. *italicum* individual growing near Vers-Pont-du-Gard, Gard department, South France (photo: Natascha Wagner). (**b**) *X. strumarium* growing at the Old Botanical Garden of the Georg-August-University of Göttingen, Germany. (**c**) Seedlings of *X. strumarium* (small) and *X. orientale* subsp. *italicum* (tall) during the competition experiments carried out in 2022. Burs were sowed in the same day, and the picture was taken approximately two weeks after the first germination. (**d**) Two trays of the allelopathy experiments carried out in 2023. Seedlings belong to the same species, the tray on the left was watered with distilled water (control), the one on the right with the leachate of the leaves of *X. orientale* (treatment). (**e**) Competition experiments in summer 2022 in the Old Botanical Garden of the Georg-August-University of Göttingen. Plants in the front are *X. strumarium* (control and intraspecific competition pots), in the back pots containing also *X. orientale* subsp. *italicum* (tall plants; Interspecific competition).

*Xanthium* L. is a genus of the subtribe Ambrosiinae (tribe Heliantheae), in the Asteraceae family^13^. The genus is characterised by the unusual pistillate inflorescences formed by two flowers enclosed completely in structures covered by hooked spines and beaks (catoclesium; hereafter also referred as bur for simplicity). Molecular marker-based studies have unequivocally demonstrated the existence of at least three distinct polymorphic species within the *X.* sect. *Xanthium*^14^: *X. strumarium* L., *X. orientale* L. and *X. chinense* Mill. The first species, *X. strumarium*, is native to the Old World, as evidenced by both fossil records^15^ and historical evidence (Dioscorides’ *De Materia Medica*, 1st Century BC). It presents small infructescences with straight apical beaks and few spines (Fig. 1b). *Xanthium orientale,* instead, is native to America but has become cosmopolitan as a result of human-assisted dispersal^14^. It is one of the most variable species complexes within the genus (Fig. 1a).

In the 1820’s, some of the first naturalised populations of *X. orientale* in Europe were detected near Pavia, in Italy, by Moretti, who described the taxon “*X. italicum* Moretti”^16^. Those populations were typically found near riverbanks and wastelands. Nowadays the species is spread throughout the Mediterranean basin, but also in other regions of the world, and is in fact considered a dangerous invasive alien weed species, causing considerable losses in agricultural crops (e.g.,^17–19^). In Europe, it is categorised as an invasive species in 20 countries, while naturalised in eight other territories^20^. Its diffusion is likely related to a high genetic diversity, high germination rates (above 90%), a long flowering period, wind pollination, zoochory and hydrochory^21^. It is not clear, however, whether the presence of *X. orientale* also affects native species and especially the native congener.

Apart from competition, allelopathy, is another important factor for plant-plant interactions. It is defined as any direct or indirect harmful or beneficial effect by one plant on another through the production of chemical compounds that escape into the environment^22,23^. The seedlings of *Xanthium*, the leaves as well as other plant parts contain poisonous alkaloids and glycosides (e.g. potassium carboxyatractyloside,^24^). Among these, many are considered allelopathic compounds, such as xanthatine, 1α, 5α-epoxyxynthatin, 4-epiisoxanthanol, 4-epixanthanol, loliolide, dehydrovomifoliol, phenolics and volatile oils^25,26^. It was proven that these chemicals do not only play an important role in plant-plant interactions (e.g.,^27–29^) but can also cause animal diseases or are even toxic for grazing animals, especially in the early growth stages of the plants ^30^.

A recent study ^31^ demonstrated a significant decline of *X. strumarium* populations in Europe following the introduction of its congeneric *X. orientale*. This was evidenced by occurrence data based on herbarium specimens of the two species dating from 1800 to the present day. A similar pattern has been observed in other parts of its original distribution range (e.g.^32–34^,). In Italy, the native species has almost disappeared from collections, with the last known collected specimen being a voucher sampled by Brullo in Sicily in 1973^31^. In contrast, the invasive species has been increasingly collected over the last century, being present nowadays in all Italian regions^35^ and considered invasive in almost half of them^36^. The decline of the native species is sometimes considered correlated to changes in land use (intensification of agriculture) and disappearance of ruderal places^37^, especially in areas where the species is traditionally considered an archaeophyte (e.g., Central and Northern Europe;^38–40^). There, the decline of the native species seems to have occurred before the arrival and spreading of the non-native congener^37^. However, studies based on fossils and pollen record have demonstrated that *X. strumarium* was already growing in natural places (e.g., riverbanks), rather than synanthropic environments in Central Europe already in prehistoric times^41^. Moreover, the hypothesis of the change in land use alone would not explain the decline of the species in areas where it is surely native and where ruderal places are still relatively abundant (e.g., The Mediterranean and the Middle East).

The factors contributing to the decline and disappearance of *X. strumarium* populations remain to be fully elucidated. The goal of the present study is to ascertain whether the interspecific competition between the European *X. strumarium* and the American *X. orientale* or the allelopathic effects of *X. orientale* over *X. strumarium* contributed to the decline of the autochthonous species populations. We conducted competition experiments in common garden and controlled climate chamber conditions to test the effect of the presence of *X. orientale* on the germination, growth and fitness of *X. strumarium* plants. The study was conducted over two years, 2022 and 2023.

## Materials and methods

### Plant material

*Xanthium* species are annual long-day herbs. In natural conditions, achenes germinate in the late spring, and the deriving plants flower in the late summer and produce mature infructescences in autumn. The two species considered in this study show slightly different phenology. *Xanthium strumarium* flowers earlier than *X. orientale*, and therefore, produces mature infructescences already in summer^14,42^.

We used *X. strumarium* burs from the Old Botanical Garden of the Georg-August University of Göttingen, where the species has been cultivated for a long time, and in summer 2022, burs were collected from a natural population growing near Kobylí (Břeclav district, Czech Republic; N 48.947050; E 16.896475). Infructescences for *X. orientale* were collected in Sicily in winter 2019–2020, in “Quattro Finaite”, Santa Flavia, on the margin of a countryside street about 15 km southeast from Palermo (N 38.037222; E 13.498889). They were collected from plants belonging to the “*X. italicum*” morphotype, which is also referred to as *X. orientale* subsp. *italicum* (Moretti) Greuter by some authors. For the experiments of 2023 (see below), we used burs collected in 2022 from plants cultivated in the botanical garden and derived from those originally collected in Italy.

### Competition experiments

We used whole infructescences (burs) and not achenes or seeds, since they are the dispersal unit of the plant and seeds germinate when they are still inside them. Burs house two fruits (achenes), each containing a single seed. In the first favourable season after ripening, only one seed germinates; the second one will germinate in the following year or afterwards^43,44^. In spring 2022, burs were sown in 3L pots containing a common well-drained soil mixture consisting of 10:3 (v/v) of a universal soil and sand, with the addition of 0.002% (v/w) Osmocote Pro fertilizer (ICL Europe B.V., Ludwigshafen am Rhein, Germany). We sowed 15 pots with only *X. strumarium* burs (control), 15 with two burs of the same species (intraspecific competition), and 20 pots containing one bur of *X. strumarium* and one of *X. orientale* (interspecific competition; Fig. 1c). Pots were placed in a YORK® climate chamber (Johnson Controls, Cork, Irland) until germination and for the first weeks after it, in order to have constant conditions (16 h of light at 25 °C, 8 h of darkness at 20 °C, 60% humidity). Germination time was registered as days after sowing. At the end of May, the pots were placed outside in the Old Botanical Garden of the University of Göttingen (Fig. 1e). Upon attaining maturity, the ripe infructescences were collected, and plants were harvested at the base of the stem and dried in a Universal Oven UF200 (Memmert GmbH, Büchenbach, Germany) for 24–36 h at 65 °C. As a proxy to measure fitness, we used the number of infructescences produced by a plant, the average weight of the infructescences and the plant dry biomass.

In 2023, we used seedlings from the control of the allelopathy experiments (see below), which were transplanted, as for the previous year, in 3L pots, and experiments followed the same scheme and conditions as in 2022.

### Allelopathy experiments

The dry *X. orientale* plants that resulted from the experiments of 2022 were used to produce leaf exudate. Dry leaves were ground and soaked in distilled water for a few days. The leachate was produced on a weekly basis. We used 100 g of leaves in 2 L of distilled water (5% w/v). The resulting exudate was then filtered and used for the allelopathy experiments.

Burs of *X. strumarium* and *X. orientale* were sown in eight trays, containing 54 0.18 L pots each. Four trays were used for *X. strumarium*, four for *X. orientale*. For each species, a total of 108 burs (two trays) were treated with leaf exudate, while 108 burs were watered with distilled water and used as a control (Fig. 1d). The pots were watered singularly every day with spray bottles. Seventeen days after the first germination, the number of seedlings was registered, and individual plants were photographed on a white background and a ruler in a HerbScan TM 008 with a Canon EOS 5SD R camera (50.6 MegaPixel; Canon Inc., Ōta, Japan). The total leaf area was extracted using the software program ImageJ v. 1.53 (^45^, available at https://imagej.net/ij/).

### Statistical analyses

After checking ANOVA assumptions, a two-way ANOVAs, followed by Tukey’s Honest Significant Difference (HSD) test for post hoc pairwise comparisons was performed to test the significance of the differences in dry biomass, numbers of infructescences produced and average weight of the infructescences of *X. strumarium* plants under a control, interspecific and intraspecific competition regime. For the intraspecific competition, the two plants growing in the same pot were both analysed separately as focal against the other. The analyses were done for both years separately. Two-ways ANOVAs and pairwise Tukey tests were also performed to test the significance of the differences in germination time and leaf area between *Xanthium* plants treated with *X. orientale* dry leaves exudate and those watered with distilled water in the allelopathy experiments conducted in 2023. All analyses were done in R v.4.4.2^46^, using the R Studio software (v.2022.02.2-485;^47^ available at http://www.rstudio.com/). Boxplots and graphics were also produced in R, using ggplot2^48^.

## Results

### Germination

**Fig. 2:**
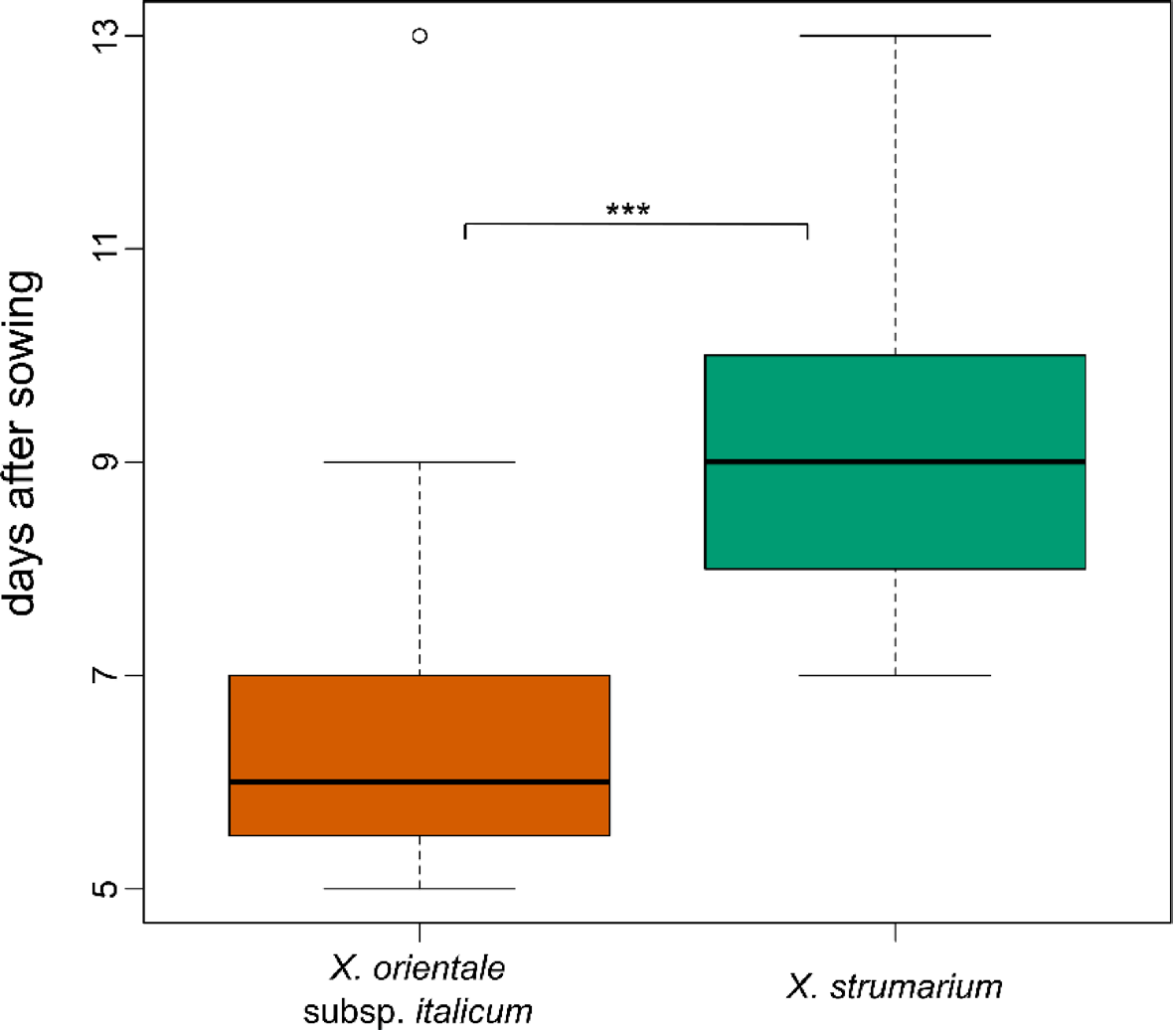
Germination time of burs of *X. strumarium* (green, second box) and *X. orientale* subsp. *italicum* (rost red, first box), measured as days after sowing. Asterisks represent statistical significance: (***) *P* < 0.001.

In the first year we tested the germination times of the two species. In general, *X. orientale* germinated sooner than *X. strumarium* (7.57 and 10.09 days after sowing on average, respectively). This difference was found to be highly significant in the ANOVA test (*p* < 0.001) (Fig. 2; Table 1).

**Table 1:** ANOVA results from the competition experiments carried out for germination confronting the two species. Asterisks represent level of significance: (***) for *P* value < 0.001, (**) *P* value < 0.01, (*) *P* value < 0.05, (.) *P* value < 0.1.

Germination					
	Df	Sum Sq	Mean Sq	F value	Pr(> F)
trat	1	123.2	123.25	47.67	1.08e^-09^***
Residuals	88	206.8	2.59		

### Competition

**Fig. 3:**
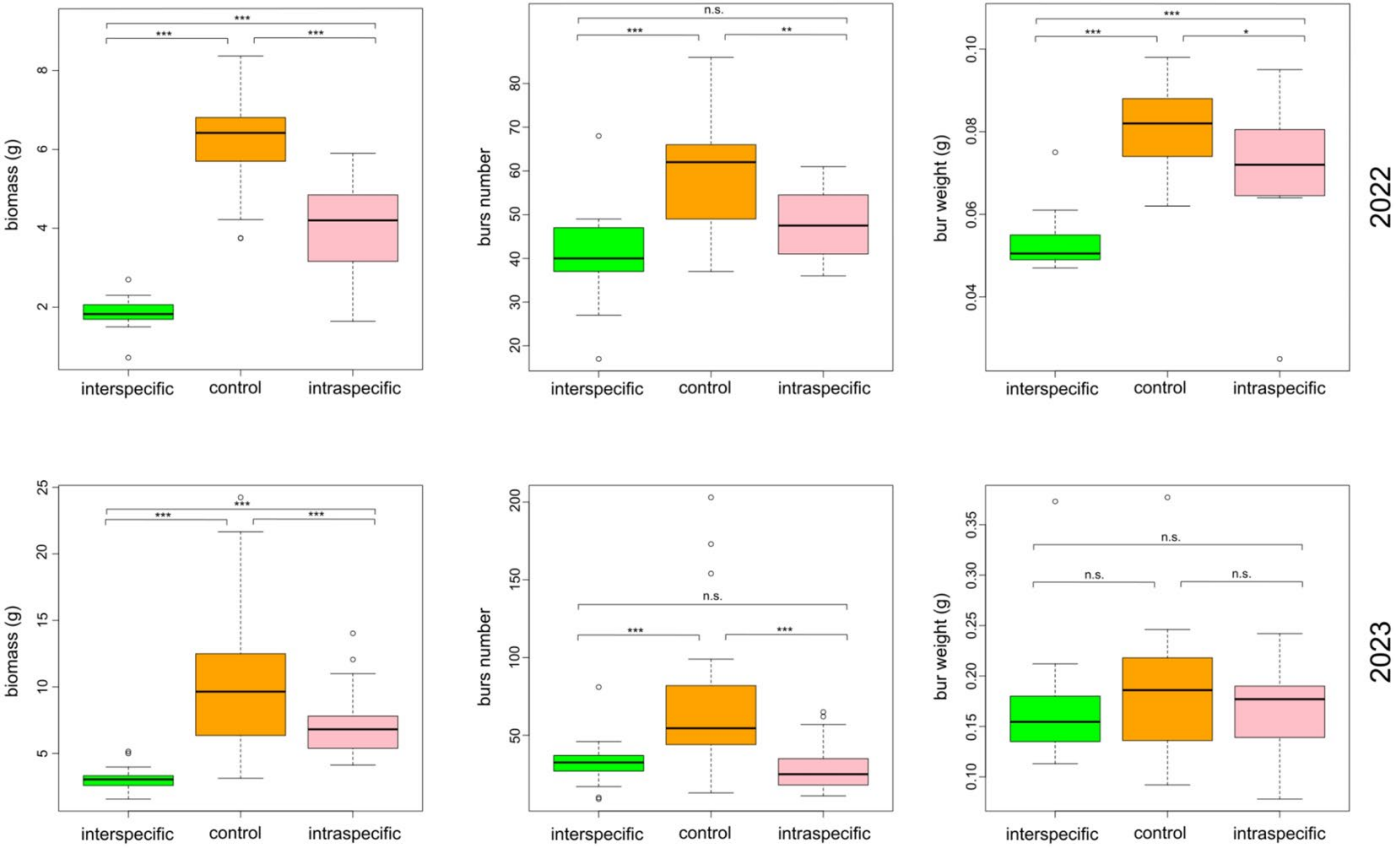
Effect of inter- (green, first box) and intra-specific competition (pink, third box) on the dry biomass, number of burs and mean bur weight of *X. strumarium*. In the two lines are shown results from the experiments of the two different years. (***) for *P* value < 0.001, (**) *P* value < 0.01, (*) *P* value < 0.05, (.) *P* value < 0.1 based on the Tukey-test.

The dry biomass of *X. strumarium* was measured from control samples and those growing in an intra- and inter-specific competition regimes. Results from the ANOVA analyses showed a significant effect of competition on biomass (Table 2) and fruit number (Table 3) of the species for the experiments carried out during the first year (*p* < 0.001 for both treatments) and the second (*p* < 0.001 for both treatments), while for the bur weight (Table 4) only in 2022 there was a significant effect (*p* < 0.001). In general, the Tukey-test (Table S1a–f) showed a significant effect of interspecific competition (*p* < 0.001) in all cases except for the bur weight in 2023, whereas for the intraspecific competition it was highly significant (*p* < 0.001) for the plant biomass in both years and for bur number in 2022, significant (*p* < 0.01) for the bur number and only marginally significant (*p* < 0.05) for the bur weight in 2022 (Fig. 3). The Tukey test analysis (Table S1a, f) also revealed a significant difference in plant biomass between the treatments (intraspecific versus interspecific competition) in both years. In 2022, a significant difference was also observed in the infructescence weight (Fig. 3; Table S1c).

**Table 2:** ANOVA results from the competition experiments carried out for plant biomass. Asterisks represent level of significance: (***) for *P* value < 0.001, (**) *P* value < 0.01, (*) *P* value < 0.05, (.) *P* value < 0.1.

		Biomass				
		Df	Sum Sq	Mean Sq	F-value	Pr(> F)
2022	*trat*	2	177.29	88.65	77.03	2.83e^−16^***
	*Residuals*	52	59.84	1.15		
2023	*trat*	2	808	404	48.34	3.72e^−16^***
	*Residuals*	121	1011	8.4

**Table 3:** ANOVA results from the competition experiments carried out for the number of infructescences produced. Asterisks represent level of significance: (***) for *P* value < 0.001, (**) *P* value < 0.01, (*) *P* value < 0.05, (.) *P* value < 0.1.

		Burs number				
		Df	Sum Sq	Mean Sq	F value	Pr(> F)
2022	*trat*	2	3469	1734.4	15.14	6.58e^−06^***
	*Residuals*	52	5957	114.6		
2023	trat	2	29,646	14,823	27.96	1.04e^−10^***
	Residuals	121	64,146	530

**Table 4:** ANOVA results from the competition experiments carried out for infructescence weight. Asterisks represent level of significance: (***) for *P* value < 0.001, (**) *P* value < 0.01, (*) *P* value < 0.05, (.) *P* value < 0.1.

		Bur weight				
		Df	Sum Sq	Mean Sq	F value	Pr(> F)
*2022*	*trat*	2	0.007188	0.003594	27.65	6.62e^−09^***
	*Residuals*	52	0.006759	0.00013		
2023	*trat*	2	0.00568	0.002842	1.55	0.216
	*Residuals*	121	0.22168	0.001832

Going more into detail, the plant biomass had a decrease of around 70.5% in 2022 and 69.7% in 2023 under the interspecific competition (*p* < 0.001) and of 34.9% and 30.8% under the intraspecific one (*p* < 0.001) for first and second year respectively. The production of infructescences was significantly influenced by the treatment, with a decrease under interspecific competition regime of 31.5% and 52.4% in 2022 and 2023 (*p* < 0.001) and of 19.8 and 57.6% under intraspecific competition (*p* < 0.01 and *p* < 0.001 for 2022 and 2023, respectively). The average bur weight, instead, was significantly affected by the treatment only in the first year, with a decrease in mass of 35.6% for interspecific treatment (*p* < 0.001) and of 12.1% for intraspecific one (*p* = 0.02). In 2023, even if not significantly, the burs mass had a decrease of 5.6 and 11.1% for the inter- and intraspecific treatments, respectively (Fig. 3; Tables 2, 3 and 4 and S1a-f).

### Allelopathy

We compared the germination times of the two species under treatment and control conditions. In general, *X. orientale* seemed to have higher germination rates. For the control individuals, 84.3% of the burs germinated for *X. orientale*, for a total of 91 seedlings, whereas 76.9% of burs germinated for *X. strumarium*, with a total of 82 seedlings.

The treatment seemed to have influenced germination of the two species, with a decrease of circa 16% of germinations for *X. orientale* (67.6% of germinated burs, for a total of 72 seedlings) and of 12% for *X. strumarium* (64.8%, for a total of 70 seedlings). However, the germination time was significantly influenced by the treatment only for *X. orientale* (*p* < 0.001 in the Tukey-test) and marginally *X. strumarium* (*p* < 0.05 in the Tukey-test; Table S1g).

**Fig. 4:**
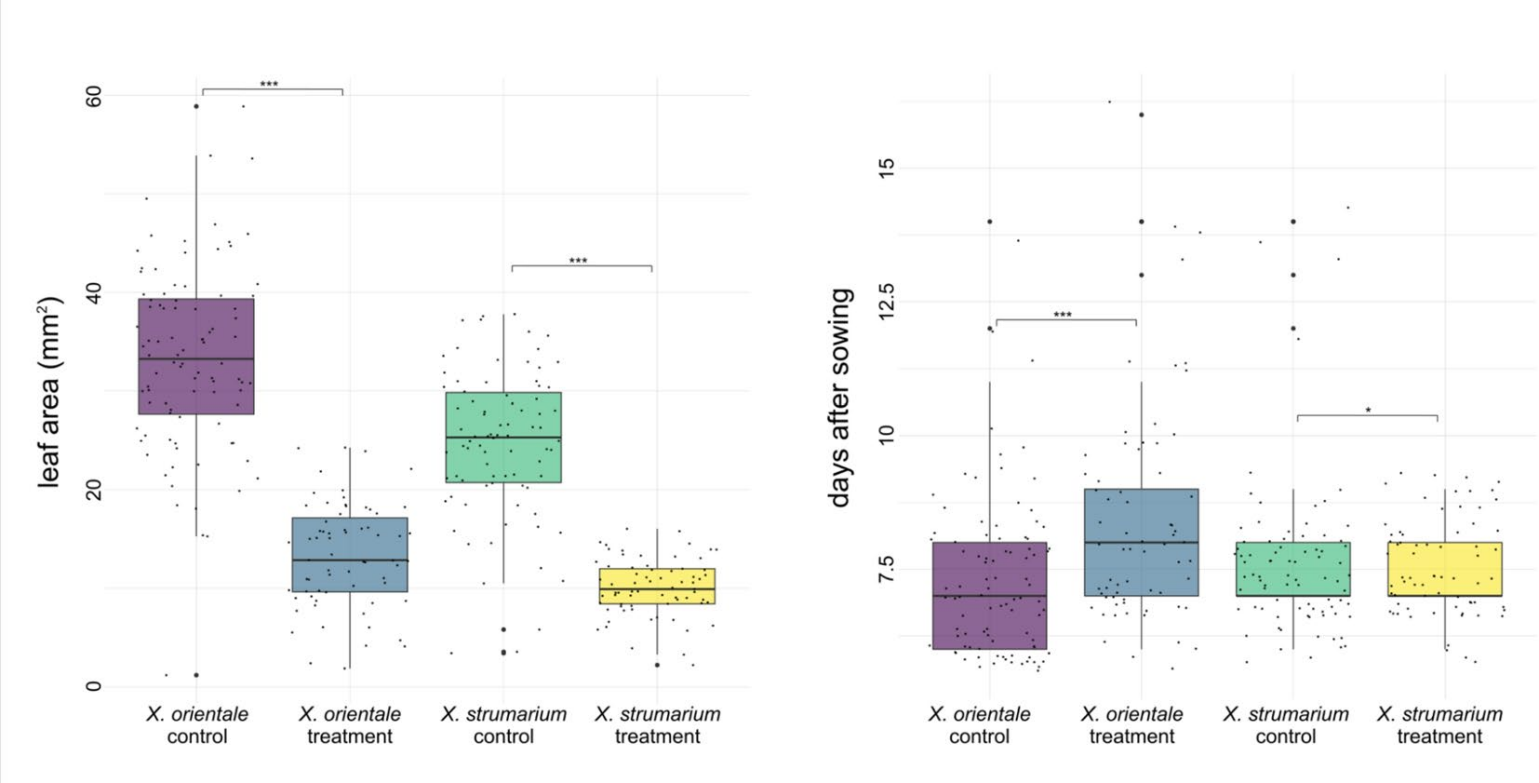
Effect of treatment with leachate of dry leaves of *X. orientale* on the germination and growth (leaves area 17 days after the first germination) of *X. strumarium* (green, third box: control; yellow, fourth box: treatment) and *X. orientale* subsp. *italicum* (purple, first box: control; caerulean, second box: treatment). (***) for *P* value < 0.001, (**) *P* value < 0.01, (*) *P* value < 0.05, (.) *P* value < 0.1.

The leaf area was significantly affected by the treatment (*p* < 0.001; Table 5). Specifically, under treatment with *X. orientale* leaves exudate, there was a reduction in growth of about the 60% in both species (*p* < 0.001; Fig. 4; Table S1h).

**Table 5:** ANOVA results from for leaf area calculated in the allelopathy experiments. Asterisks represent level of significance: (***) for *P* value < 0.001, (**) *P* value < 0.01, (*) *P* value < 0.05, (.) *P* value < 0.1.

Leaf area					
	Df	Sum Sq	Mean Sq	F value	Pr(> F)
trat	3	27,180	9060	185.80	< 2e^−16***^
Residuals	306	14,923	49

## Discussion

In the present study, garden experiments were conducted over a period of two consecutive years in order to ascertain whether the presence of the introduced *X. orientale* could be considered a contributing factor to the observed decline of *X. strumarium* populations in the Old World.

Invasive alien species can cause the displacement and local extinction of native congeners through various mechanisms, with the most significant being habitat destruction, competitive exclusion, reproductive interference, and hybridisation. We tested competitiveness of the American *X. orientale*, nowadays widespread in different parts of the world, with the one of the congeneric *X. strumarium*, native to the Old World and declining in different parts of its distribution range. In both years, the competition with *X. orientale* was observed to have a detrimental effect on the growth and reproduction of *X. strumarium*. The reduction in biomass, and thus growth, of the plants growing under interspecific competition, along with the lower number of ripened fruits, may suggest that the competition of the congener is one of the factors causing the decrease of *X. strumarium* populations over the years with, consequently, the almost complete disappearance of the species in its native range. Alien plant species are considered to be a contributing cause of about 25% of plant extinctions^49,50^. Trophic competition has been often indicated as cause of exclusion of native species by introduced invaders, even though evidence supporting a general and primary role of the invasive aliens in the displacement of the native species remains limited^51^. In most studies on plants reviewed by Gurevitch and Padilla^51^, competition and indirect habitat effects of alien plants were believed to be the main threats causing population declines of native species. The deleterious effect of competition for the co-existence of native and alien species is supposed to be more important in closely related species (e.g., congeners). This principle, also known as “competition-relatedness hypothesis”^52^ was first formulated by Darwin^4^ and later discussed by several researchers^53–55^. However, only a few studies have corroborated these expectations on experimental ground, both in plants ^10,81^  and in animals^12^.

It is noteworthy that, although patterns are generally consistent, differences are registered in the mean biomass, number and mean weight of the infructescences between the two years. In the first year, plants of *X. strumarium* were on average smaller, and produced fewer and smaller (lighter) burs. Summer 2022 was extraordinarily dry in Western and Central Europe, which is probably the reason for these differences. This is also reflected in the effect of the competition of *X. orientale* on *X. strumarium*, as observed in the difference of infructescence weight between controls and plants growing under interspecific competition, which was significant only in 2022. Probably, the better climatic conditions of 2023 mitigated competition, suggesting that the effect of nter- and intra-specific competition can differ considerably under different environmental conditions, as observed already in other plant groups^56^.

Another important factor influencing plant interactions is allelopathy. It can act in combination with resource competition influencing growth and fitness of plant species growing in sympatry^57^, as demonstrated by empirical studies on tree species^58^. In our experiments, a lesser number of seedlings germinated under treatment with the leachate of the leaves of *X. orientale* compared to the control, both in *X. strumarium* and in *X. orientale*. The leachate negatively influenced the growth of the seedlings of both species. If, on the one hand, these results confirmed the allelopathic effect of *X. orientale* on the germination and growth of other species, as already observed in numerous other studies (e.g.,^27–29^), on the other hand, they evidenced an allelopathic effect of a species over itself, which is something rarely tested in other studies. The fact that we found a significant effect of the leachate of *X. orientale* on the germination and growth of seedlings of the same species opens questions on the significance of such experiments and on the biological meaning of the results. In our particular case, it could be hypothesized that burs of *X. orientale* germinate earlier, and seedlings grow faster than those of the congener (see Fig. 1) and start inhibiting via allelopathy and later on via resource competition the growth and reproduction of those plants germinating later (i.e., *X. strumarium*).

However, it must be noticed that both species grow in relatively open environments (seashores, riverbanks, ruderal places), where the effect of competition might be less important in determining population dynamics of both species. It is therefore questionable if competition and allelopathy alone are enough to explain the pattern of displacements observed in *X. strumarium* in the Old World. Another important mechanism that can produce the displacement of one species by a congener is Reproductive Interference (RI; i.e., the negative reproductive interaction among closely related species). RI can often impede coexistence of close relatives^59,60^. In plants, RI can be generated by various mechanisms, e.g., loss of ovules through seed set failure, pollen allelopathy, stigma clogging caused by interspecific pollen transfer, and the production of unviable hybrids (see^61^ and reference therein). The presence of asymmetric RI was already found in the genus *Xanthium*, in empirical studies involving *X. chinense* and *X. orientale* (*X. orientale* subsp. *italiucum*) in Japan^32^. However, it must be noticed that, differently from the two above-mentioned species, *X. strumarium* has a different phenology than *X. orientale*, which precludes any overlap of the flowering time between the two species^14^. In our garden experiments, burs of *X. strumarium* ripened well before *X. orientale* plants began flowering. Moreover, self-pollination can mitigate the negative effects of mutual reproductive interference between coexisting congeners^62^, and *Xanthium* is very well known to have a mating system promoting selfing^63^, as also corroborated by studies based on isozyme data^64^. Therefore, it seems unlikely that RI plays an important role in this setup, although experimental studies, using ecotypes of *X. orientale* more adapted to cold environments (and therefore flowering earlier;^65,66^), may help addressing the issue.

An alternative way in which a close relative invader can cause local extinction in native plant species is via hybridiszation, in cases where the formed hybrids are viable and can backcross with the parental species^67^. Hybridiszation with invasive congeners can threaten rare native taxa to extinction via genetic swamping, where the rare form is progressively replaced by hybrids, or by demographic swamping due to wasteful production of maladaptive hybrids (the former being more frequent;^68^). In such cases, extinction can occur within a few generations^69^ and numerous examples have been observed in different plant species^70–73^. Although most studies focused on hybrid extinction of rare species^74,75^, simulation studies demonstrated that even common species are at risk when exposed to highly competitive and invasive congeners, especially if hybrids are also competitive and highly fertile^76^. However, in our specific case, the aforementioned difference in flowering time should preclude the hybrid formation. Phenological differences hindering hybridiszation between different lineages in *X.* sect. *Xanthium* was already reported in other studies^65^. McMillan ^82^ was able to produce artificial hybrids between *X. strumarium* and *X. orientale*, which however were not highly fertile. The relative infertility of the experimental hybrids was interpreted to be a strong enough barrier to prevent the production of hybrid swarms and therefore maintain the genetic identity of *X. strumarium*^77,78^. In phylogenomic analyses including several samples of the two species^79^, no evidence of hybridiszation was found, although putative hybrids between the two species were included in the analyses (e.g., the original material of “*X. sallentii* Sennen” and “*X. widderii* Sennen”;^80^).

In conclusion, the presence of *X. orientale*, invasive to a vast part of the Old World, negatively influences the growth of the native congener *X. strumarium* via resources competition and allelopathy. This could have contributed to the decline and disappearance of *X. strumarium* observed in a great part of its distribution range. However, other potential causes, such as the disappearance of some of the suitable habitats (ruderal and waste places, extensively cultivated fields), the dispersal agents (e.g., the decline of transhumant sheep cattle in part of Europe), may have also played an important role on the decline of *X. strumarium*, at least in part of its former distribution range. The extent to which those processes contributed, in alternative or in combination with the biotic factors taken into account in our study, needs to be further investigated.

## Supplementary Information

Below is the link to the electronic supplementary material.

Supplementary Material 1

## Data Availability

The datasets used and/or analysed during the current study are available from the corresponding author on reasonable request.
